# The rise and fall of an ancient Adélie penguin ‘supercolony’ at Cape Adare, Antarctica

**DOI:** 10.1098/rsos.172032

**Published:** 2018-04-18

**Authors:** Steven D. Emslie, Ashley McKenzie, William P. Patterson

**Affiliations:** 1 Department of Biology and Marine Biology, University of North Carolina, 601 South College Road, Wilmington, NC 28403, USA; 2 Saskatchewan Isotope Laboratory, 114 Science Place, Saskatoon, Saskatchewan, Canada S7N 5E2

**Keywords:** Ross Sea, *Pygoscelis adeliae*, stable isotopes, sea level rise, population movement

## Abstract

We report new discoveries and radiocarbon dates on active and abandoned Adélie penguin (*Pygoscelis adeliae*) colonies at Cape Adare, Antarctica. This colony, first established at approximately 2000 BP (calendar years before present, i.e. 1950), is currently the largest for this species with approximately 338 000 breeding pairs, most located on low-lying Ridley Beach. We hypothesize that this colony first formed after fast ice began blocking open-water access by breeding penguins to the Scott Coast in the southern Ross Sea during a cooling period also at approximately 2000 BP. Our results suggest that the new colony at Cape Adare continued to grow, expanding to a large upper terrace above Ridley Beach, until it exceeded approximately 500 000 breeding pairs (a ‘supercolony’) by approximately 1200 BP. The high marine productivity associated with the Ross Sea polynya and continental shelf break supported this growth, but the colony collapsed to its present size for unknown reasons after approximately 1200 BP. Ridley Beach will probably be abandoned in the near future due to rising sea level in this region. We predict that penguins will retreat to higher elevations at Cape Adare and that the Scott Coast will be reoccupied by breeding penguins as fast ice continues to dissipate earlier each summer, restoring open-water access to beaches there.

## 1. Introduction

**Figure 1.**
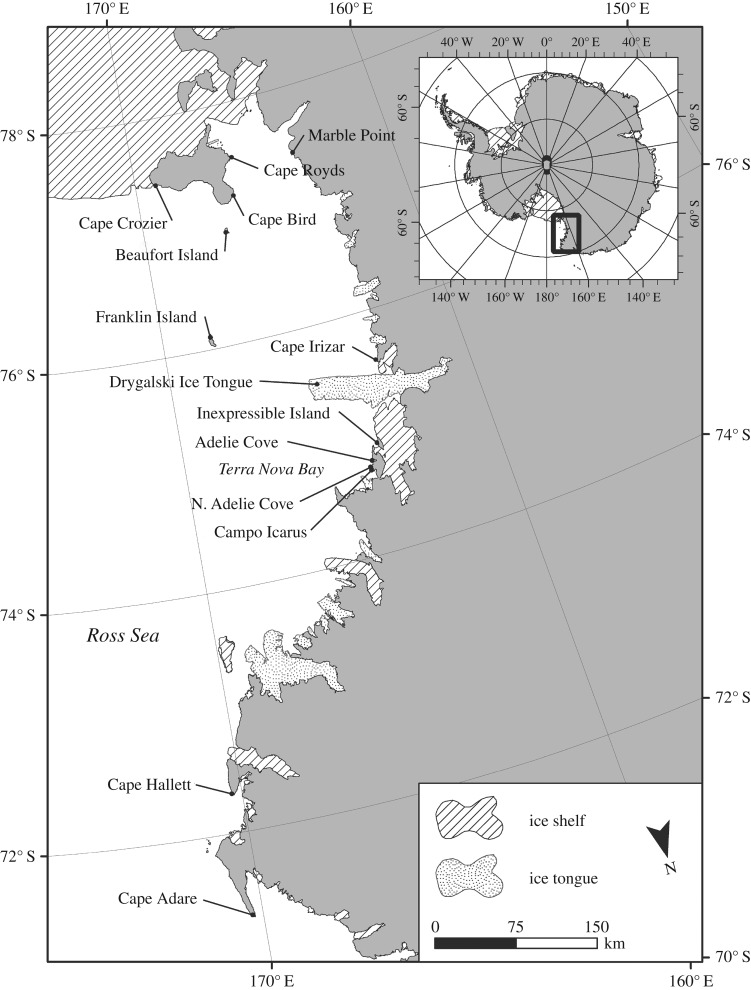
Map of the Ross Sea giving locations of active and abandoned Adélie Penguin colonies discussed in the text. Note location of Cape Adare and Cape Hallett at the entrance to the Ross Sea to the north.

The Adélie penguin (*Pygoscelis adeliae*) is one of only two endemic species of penguin in Antarctica; it is circum-Antarctic in distribution and numbers in the millions [1]. This species also is distributed in a manner with several very large colonies (greater than 100 000 breeding pairs) at key locations, with smaller colonies distributed nearby [2]. Most colonies are located near coastal polynyas (areas of persistent open-water surrounded by sea ice), which provide open water access to breeding sites as well as highly productive marine food webs in the marginal ice zones [1,3]. The consistently largest colony in Antarctica is located at the entrance to the Ross Sea at Cape Adare (figure 1), where population estimates have ranged from 220 900 to 282 307 breeding pairs in the 1980s [4]. The population declined to 169 200 breeding pairs in the 1990s followed by a large increase with the current population estimated at 227 000 in 2012 (from colony counts) [5] and 338 231 nesting pairs in 2014 based on satellite imagery [6].

**Figure 2.**
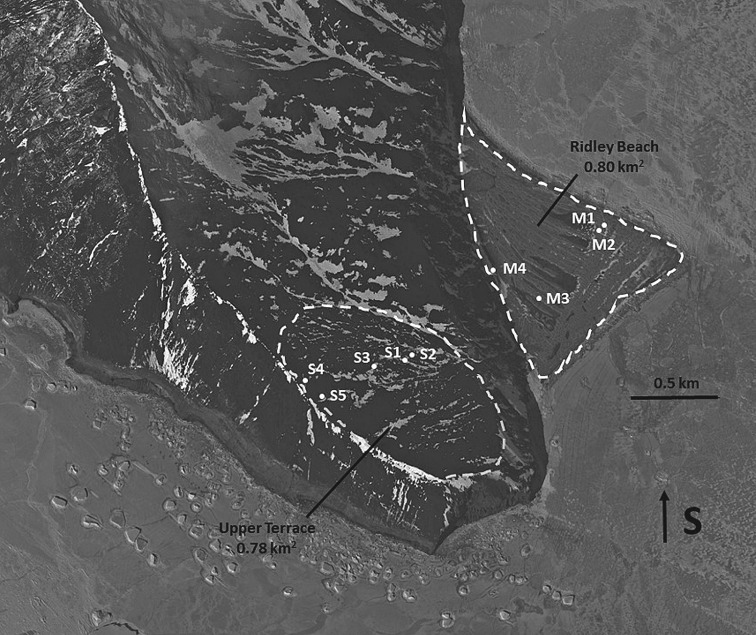
Google Earth image of Cape Adare with locations of sampling sites indicated as either mounds (M) on Ridley Beach or sites (S) on the upper terrace. Note the parallel ridges on Ridley Beach that are entirely composed of ornithogenic soils deposited over the past 2000 years. Map data: Google, DigitalGlobe 2017.

Most nesting penguins at Cape Adare are located on long, parallel ridges or mounds that cross Ridley Beach, a large triangular beach with an area of approximately 0.8 km^2^ (figure 2). Previous excavations of these ridges have revealed that they are composed entirely of ornithogenic soils, indicating that they are all penguin-formed with natural beach sands at the base [7]. With the beach completely covered with nesting penguins, additional penguins have nesting sites that extend 300 m up a steep slope to a large upper terrace that extends to the south and southwest. A few small active subcolonies are located at the edge of this terrace overlooking Ridley Beach. We first investigated Ridley Beach and this upper terrace in January 2005 and completed excavations at active and abandoned penguin mounds to determine the age and extent of this large colony. At that time, our surveys were limited to a central area of the upper terrace, where numerous abandoned sites were located. Three of these sites (sites S1–S3; figure 2) were sampled with excavations. We also sampled four of the mounds associated with active subcolonies on Ridley Beach (mounds M1–M4; figure 2) and determined from radiocarbon analyses that this beach has been continuously occupied by breeding Adélie penguins for at least the past approximately 2000 years [7]. The upper terrace was colonized slightly later than the beach at approximately 1700 BP, indicating that this terrace was not occupied by breeding penguins until after all potential nest sites on the beach were taken.

In January 2016, we revisited the upper terrace at Cape Adare to conduct additional surveys and sampling. Though ground time was limited owing to rapidly changing weather conditions, we were able to locate numerous other abandoned penguin sites that extend to the south edge of this terrace. Further, these sites all had dry, ancient ornithogenic soils indicating none had been occupied recently. Two such sites were sampled with excavations, the most distant being approximately 1 km south of the terrace edge overlooking Ridley Beach. Here, we report new radiocarbon dates from these excavations, as well as additional dates on samples collected during the 2005 excavations, and present evidence that the entire Cape Adare colony was once nearly twice the size that it is today with occupation of most of the upper terrace by approximately 1200 BP. We also use stable isotope analysis of modern penguin egg membrane to determine if dietary differences among penguin colonies in the Ross Sea help explain the large occupation at Cape Adare, past and present.

## 2. Methods

### 2.1. Excavations and sampling

Abandoned mounds and subcolonies were mapped using a handheld Garmin GPSMAP 78s. Locations of sites (lat./long.) were imported into Google Earth Pro (v. 7.3.0). Area measurements in square kilometres were obtained by using the polygon tool in this software. Nine sites were excavated and/or sampled following previously published methods in [8] during both visits to Cape Adare in January 2005 and 2016. At each sampling site, a 1 × 1 or 0.5 × 0.5 m test pit (size of pit varied based on surface conditions and time available in the field) was placed at the centre of the abandoned subcolony, or the sites were probed and sampled with a trowel. Surface pebbles were removed and placed on a tarpaulin. Excavations proceeded in 5 cm levels with all excavated sediments dry-screened through two nested screens with mesh sizes of 0.64 and 0.32 cm^2^, respectively. Organic remains were separated from the larger mesh screen in the field and sediment from the 0.32 cm^2^ mesh screen was placed into a large sediment bag by level and saved for additional analysis and sorting in the laboratory. Excavations continued until the bottom of ornithogenic soils was reached as recognized by a change in colour and texture of the soil. The pits were then backfilled and all surface pebbles were replaced. These methods were used on three sites (S1–S3) on the upper terrace in 2005 with two additional sites (S4 and S5) excavated in 2016 (figure 2).

Penguin mounds on Ridley Beach in 2005 were too deep and extensive to sample using these methods. Instead, one mound (M3) was found eroded through its centre, exposing a greater than 1 m deep profile of the entire mound. It was sampled by cleaning the profile and obtaining organic remains (penguin bones, feathers) from the upper, middle and lower layers, including the bottom interface where volcanic beach gravels and sand indicated the base of the ornithogenic deposits. Three other mounds (M1–M2, M4; figure 2) were sampled by excavating small holes with a hand trowel to probe to the base of the mounds and obtain additional organic remains for analysis.

In 2005, five recently hatched penguin eggshell samples were collected by active subcolonies located at the edge of the upper terrace overlooking Ridley Beach. Sampling activities in 2016 were restricted to areas away from these active subcolonies and no additional modern eggshell samples were collected. However, we collected recently hatched eggshell at three other active Adélie penguin colonies farther south in the Ross Sea in 2016 at Cape Hallett, Adélie Cove and Inexpressible Island (figure 1).

### 2.2. Radiocarbon analysis

Eight radiocarbon dates were completed on samples collected in 2005 by Beta Analytic, Inc., and are reported in [7]. Seven additional radiocarbon dates were completed on eggshell from M2 sampled in 2005 (two dates), and from feather, eggshell and egg membrane from S4 to S5 sampled in 2016. These seven samples were submitted to the Woods Hole radiocarbon laboratory (NOSAMS) for accelerator mass spectrometry dating and are reported with NOSAMS sample numbers. Each of the 15 radiocarbon dates (in radiocarbon years before present, BP) was corrected and calibrated for the marine carbon reservoir effect using Calib 7.1 and the Marine13 calibration curve [9–10] with a Δ*R* = 750 years and are reported here in calendar years BP. This calibration provided a 2-*σ* range and median age for estimating the true age of each sample.

### 2.3. Stable isotope analysis

Stable isotope analysis of carbon and nitrogen from modern penguin egg membrane was completed at the Stable Isotope Laboratory, University of Saskatchewan, Saskatoon, Canada. Stable isotope values were obtained using a Thermo Finnigan Flash 1112 EA coupled to a Thermo Finnigan Delta Plus XL via a Conflo III. Carbon isotope ratios are reported in per mil notation relative to the VPDB scale. Nitrogen isotope ratios are reported in per mil notation relative to AIR. Carbon isotope data are calibrated against the international standards L-SVEC (*δ*^13^C = −46.6‰ VPDB) and IAEA-CH6 (*δ*^13^C = −10.45‰ VPDB). Nitrogen data are calibrated against the international standards USGS-25 (*δ*^15^N = −30.4‰ AIR) and IAEA-305A (*δ*^15^N = 39.8‰ AIR). Precision of *δ*^13^C and *δ*^15^N measurements are 0.12‰ and 0.22‰, respectively (*n* = 18, 2*σ*). %C and %N measurements have a precision of ±10% of the reported percentage. We used one-way ANOVA and a Shapiro–Wilk's normality test using Sigmaplot 13 (Systat Software, Inc.) to test for differences in stable isotope values in egg membrane among the four penguin colonies.

## 3. Results

### 3.1. Radiocarbon dates and occupation at Cape Adare

The seven new radiocarbon dates on penguin tissues collected in 2005 and 2016 produced calibrated calendar ages ranging from 785 to 1386 BP (median ages, table 1) except for one date on a feather from S4 which was too young in age for calibration and essentially modern. Increasingly, younger dates occurred with sites on the upper terrace above Ridley Beach and more distant to the south and southeast. The oldest date from Cape Adare, first reported in [7] from the base of ornithogenic soils exposed at M3 on Ridley Beach, has a median age of 1962 BP (table 1). The youngest median dates reported here are from M4 middle sediment (780 BP) and S4 level 1 (785 BP). S4 is located approximately 1 km southeast of the edge of the upper terrace where active penguin colonies are currently located and is the most distant site from the water so far discovered at Cape Adare (figure 1).

**Table 1.** Radiocarbon dates on Adélie penguin tissues from ornithogenic soils at Cape Adare, Antarctica. (Uncorrected dates are in radiocarbon years before present (BP); dates were corrected for the marine carbon reservoir effect and calibrated with the Marine13 calibration curve using Calib 7.1 [9,10] and a Δ*R* = 750 ± 50 years to provide 2-sigma ranges and median dates in calendar years BP. Absence of 2-sigma values are dates that were too young for calibration and essentially modern in age. All dates with OS laboratory numbers are samples collected in 2016 and were analysed at the Woods Hole National Ocean Sciences Accelerator Mass Spectrometry (NOSAMS) facility; dates with Beta laboratory numbers are from samples collected in 2005 and analysed at Beta Analytic, Inc., Coral Gables, Florida, and were previously reported in [7] and recalibrated using the newer version of Calib (Calib 7.1).)

laboratory no.	location	material	uncorrected ^14^C age	calibrated 2-sigma range	median
*Ridley Beach*					
OS-139987	mound 2, lower sediments	eggshell	2580 ± 20	1506–1277	1376
OS-139988	mound 2, lower sediments	eggshell	2590 ± 15	1510–1284	1386
Beta 202965	mound 3, lower sediments	eggshell	3090 ± 40	2128–1808	1962
Beta 202966	mound 3, lower sediments	eggshell	3000 ± 40	2019–1691	1856
Beta 202967	mound 4, middle sediments	chick bone	1980 ± 60	924–646	780
Beta 202968	mound 4, lower sediments	chick bone	2470 ± 40	1405–1136	1274
Beta 202969	mound 4, lower sediments	eggshell	2430 ± 50	1368–1069	1234
*Upper Terrace*					
Beta 202972	site 1, lower sediments	eggshell	2140 ± 40	1088–778	938
Beta 202971	site 2, lower sediments	chick bone	2890 ± 50	1888–1547	1727
Beta 202970	site 3, lower sediments	chick bone	2840 ± 60	1865–1491	1668
OS-136459	site 4 Lev 1	feather	790 ± 20	—	
OS-139990	site 4 Lev 1	eggshell	1990 ± 15	898–679	785
OS-139991	site 4 Lev 1	eggshell	2440 ± 40	1371–1099	1245
OS-136458	site 5 Lev 1	eggshell	2320 ± 20	1252–995	1130
OS-139989	site 5 Lev 1	egg mem.	2330 ± 20	1259–1006	1141

### 3.2. Stable isotope data

**Figure 3.**
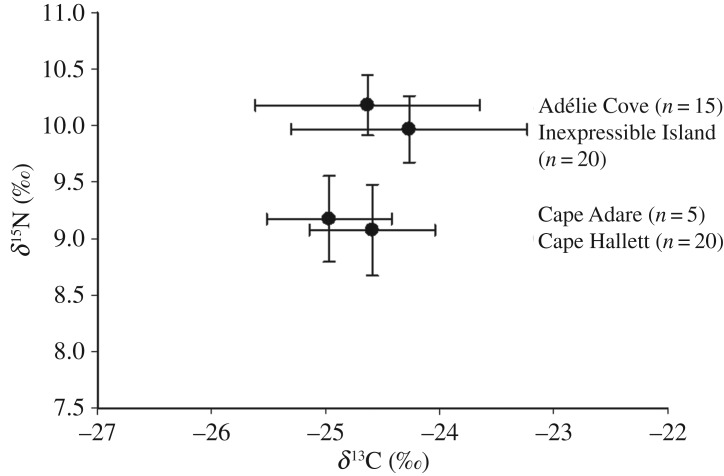
Mean *δ*^13^C and *δ*^15^N isotope values from modern Adélie penguin egg membrane collected from four active penguin colonies in the Ross Sea. Samples from Cape Adare were collected in January 2005; all other samples were collected in January 2016. Error bars are one standard deviation. See the electronic supplementary material, table S1 for raw data.

Stable isotope data (electronic supplementary material, table S1) indicate that Adélie penguins at Cape Adare and Cape Hallett, located approximately 100 km to the south, had significantly lower *δ*^15^N values in egg membrane than those from active colonies located farther to the south at Adélie Cove and Inexpressible Island at Terra Nova Bay (ANOVA, *F*_3_ = 41.8, *p* < 0.001; table 2 and figure 3). There was no difference in *δ*^15^N values between Cape Adare and Cape Hallett, or between Adélie Cove and Inexpressible Island. No differences in *δ*^13^C values occurred among all four sites (ANOVA, *F*_3_ = 1.2, *p* > 0.32).

**Table 2.** Mean ± s.d. of stable isotope results of modern Adélie penguin egg membrane from four breeding colonies in the northern to central Ross Sea, Antarctica. (For each set of isotope values, shared letters (in superscript) indicate no significant differences in means (one-way ANOVA, *F*_3_ = 1.2, *p* < 0.32 for *δ*^13^C, *F*_3_ = 41.8, *p* < 0.001 for *δ*^15^N).)

location	*N*	*δ*^13^C (‰)	*δ*^15^N (‰)
Cape Adare	5	−25.0 ± 0.55^A^	9.2 ± 0.38^A^
Cape Hallett	20	−24.6 ± 0.55^A^	9.1 ± 0.40^A^
Adélie Cove	15	−24.6 ± 0.98^A^	10.2 ± 0.27^B^
Inexpressible Island	21	−24.3 ± 1.04^A^	10.0 ± 0.30^B^

## 4. Discussion

Our results suggest that Cape Adare, first occupied by breeding penguins at approximately 2000 BP [7] follows an expected colonization pattern with Ridley Beach completely occupied by approximately 1200 BP and remaining so today. Colonization of the upper terrace began by approximately 1700 BP with the most distant colonies to the south on this terrace occupied by approximately 1200 BP (based on median ages, table 1), with at least some remaining active until approximately 800 BP. The relative brief occupation of these sites also is suggested by their relatively shallow (one level, or 5–8 cm) ornithogenic soils. Only the northern edge of this terrace overlooking Ridley Beach remains occupied today.

Numerous other abandoned pebble mounds were located on the upper terrace at Cape Adare, but ground time was too limited in 2016 for additional sampling. We believe all of these sites, owing to their similar appearance on the surface, were colonized during the same sequence of occupation as S1–S5. If so, the large area of occupation on this terrace (approx. 0.78 km^2^, or similar to area on Ridley Beach at 0.80 km^2^; figure 2) is conservatively estimated to have supported an additional approximately 200 000 breeding pairs of Adélie penguins at peak occupation. Thus Cape Adare, though currently one of the largest Adélie penguin colonies in Antarctica, was possibly up to twice as large by approximately 1200 BP than it is today and consisted of approximately 500 000 breeding pairs at that time. We hypothesize that this ‘supercolony’ (a penguin colony with greater than 500 000 nests) underwent continuous growth after initial colonization at approximately 2000 BP until approximately 800 BP when it began declining to its present size.

What factors were driving the increase in this penguin ‘supercolony’ during this period? Other events in the Ross Sea at that time may help explain this hypothesized growth at Cape Adare. From 4000 to 2000 BP, the Scott Coast as well as other locations on Beaufort and Franklin Island were occupied by breeding penguins during a warm period known as the penguin ‘optimum’ [7,11,12]. The Scott Coast was completely abandoned after 3000–2000 BP, with the youngest site at Marble Point (figure 1), probably owing to a cooling period that caused increased fast ice that blocked access to beaches along this coastline, preventing penguins from breeding there [12]. This fast ice in western McMurdo Sound persists well into the summer months today and the Scott Coast has remained abandoned by breeding penguins to the present. We hypothesize that colonization at Cape Adare began as the Scott Coast was being abandoned by approximately 2000 BP, signifying a large-scale movement of breeding penguins from the southern to the northern Ross Sea. Further, upwelling of Circumpolar Deep Water in the northern Ross Sea from the continental shelf break has maintained open water in the Ross Sea polynya near Cape Adare, along with the high marine productivity at the marginal ice zone [13,14]. The northern Ross Sea and associated polynya have provided and continue to support large swarms of krill that in turn support large populations of breeding penguins that currently occur at Cape Adare, Cape Hallett (61 160 nests) and Coulman Island (19 437 nests) [6]. Though Cape Adare has been ice-free for thousands of years [15], Ridley Beach may not have been accessible to breeding penguins prior to 2000 BP owing to its low elevation, causing it to be either submerged at slightly higher sea level or too exposed to storm surges in the warming phase of the penguin optimum. Once the beach did become accessible, it was able to support increasing numbers of breeding penguins, especially with development of the higher-elevation ornithogenic mounds that currently transect the beach. Though the upper terrace could have been occupied prior to 2000 BP, older ornithogenic deposits have yet to be discovered and additional investigation is warranted.

Stable isotope data also indicate that penguins at Cape Hallett and Cape Adare presently feed significantly more on krill than fishes, as indicated by lower *δ*^15^N values in egg membrane from these sites compared to similar samples from active colonies in the Terra Nova Bay and southern Ross Sea regions. Although the sample size of modern egg membrane from Cape Adare is small (*n* = 5), these results support an earlier study on modern penguin eggshell *δ*^13^C and *δ*^15^N from colonies in the southern Ross Sea compared with Cape Hallett to the north that indicated a more krill-based diet at this latter site [16]. While these studies on eggshell and membrane only represent diet of female penguins prior to egg laying, other dietary studies at modern and ancient colonies support these dietary differences between northern and southern colonies in the Ross Sea. For example, investigation of Adélie penguin diet at Cape Hallett, based on stomach flushing and contents [17], found that these penguins feed largely on krill during the guard stage of chick-rearing and prey increasingly on fishes as the season progresses. Satellite tracking of foraging penguins also revealed that most move to the continental shelf break. Moreover, stable isotope analyses of ancient penguin guano also suggest a diet based more on fishes in the southern Ross Sea during the Holocene [18] that persists with colonies on Ross Island today (colonies at Cape Royds, Bird and Crozier, figure 1) [19]. Prey remains and otoliths from ornithogenic soils excavated at abandoned colonies on Ross Island also indicate that Antarctic silverfish (*Pleuragramma antarcticum*) has been a major component of penguin diet there for at least the past millennium [20].

Given the high marine productivity and krill availability in the northern Ross Sea today, which factors caused a decline in penguins at Cape Adare after the ‘supercolony’ reached its maximum extent by approximately 1200 BP? We have no explanation for this decline except that it was probably associated with changes in wind patterns, air temperatures, and the size of the Ross Sea polynya that ultimately affected marine productivity [21]. Alterations of this nature have caused total breeding failure, lowered chick production and high mortality events in Adélie penguins in the Ross Sea and East Antarctica [22,23] and continuing impacts over geological time could result in colony decline or abandonment. The relatively brief occupation indicated by the shallow ornithogenic soils that characterizes S4–S5 on the upper terrace supports this conclusion.

Ridley Beach remains fully occupied at Cape Adare today, but the beach remains at or near sea level with occasional flooding from storm surges and winds, as indicated by the pools of standing water in low areas between the ornithogenic ridges. Glacial melt and climate warming now occurring in Antarctica are causing sea level to rise at an enhanced rate of at least 2.0 ± 0.8 mm per year above the mean for oceans south of 50° S [24]. Moreover, average summer temperatures have been increasing by 0.5°C per decade at McMurdo Station since the 1980s [25]. We believe that the penguin colony on Ridley Beach is thus highly endangered and probably will be abandoned owing to sea level rise and increased impacts of storms and storm surges on nesting penguins. As this beach becomes uninhabitable, it is conceivable that penguins will seek higher ground and again begin to occupy former colonies on the upper terrace, a situation currently taking place at Beaufort Island [25]. Cape Hallett also is on a large beach at or near current sea level and will probably be abandoned with future sea level rise as well, but there are no higher-elevation terraces at that location for breeding penguins to retreat to. Thus, gradual displacement of hundreds of thousands of breeding Adélie penguins can be expected in the northern Ross Sea if current warming trends and rates of sea level rise continue at their current pace. The abandonment of Cape Adare and Cape Hallett could also result in a reverse of the large-scale population movements that occurred at approximately 2000 BP. The current warming trends are causing more frequent breakouts of the fast ice blocking the Scott Coast in summer each year [26] and this ice will eventually disappear, allowing breeding penguins access to beaches and former breeding sites that have remained unoccupied for the past 2000 years.

Penguin occupation in the Ross Sea continues to be a dynamic process with new colonies forming and others abandoned over geological time with changes in sea ice conditions, access to breeding sites and climate change [7,27,28]. What we are witnessing today in the Ross Sea is an example of how penguins have responded to climate change over millennia, except that it is occurring at a faster pace as warming trends and sea level rise accelerate in this region.

## Supplementary Material

Stable isotope raw data on penguin egg membrane

## Data accessibility

The results of all isotope analyses are presented in the electronic supplementary material.
